# Supplementary material for: Cooperative control of a DNA origami force sensor
Source: Sci Rep. 2024 Feb 19;14:4132. doi: 10.1038/s41598-024-53841-3 (PMC10876929; doi:10.1038/s41598-024-53841-3)
Supplement: Supplementary file 1 — Supplementary Information. [file 41598_2024_53841_MOESM1_ESM.pdf]

Supplementary Information:

Cooperative control of a DNA origami force sensor

Ariel Robbins[1], Hazen Hildebolt[2], Michael Neuhoff[2], Peter Beshay[3], Jessica O. Winter[1,4,5], Carlos E. Castro[1,3], Ralf Bundschuh[1,2,6,7], and Michael G. Poirier[1,2,6]*

[1]Biophysics Graduate Program, [2]Department of Physics, [3]Department of Mechanical and Aerospace Engineering, [4]William G. Lowrie Department of Chemical and Biomolecular Engineering, [5]Department of Biomedical Engineering, [6]Department of Chemistry and Biochemistry, and [7]Division of Hematology, Department of Internal Medicine, The Ohio State University, Columbus, OH 43210, USA.

*To whom correspondence should be addressed. Tel: 614-247-4493, Email: poirier.18@osu.edu

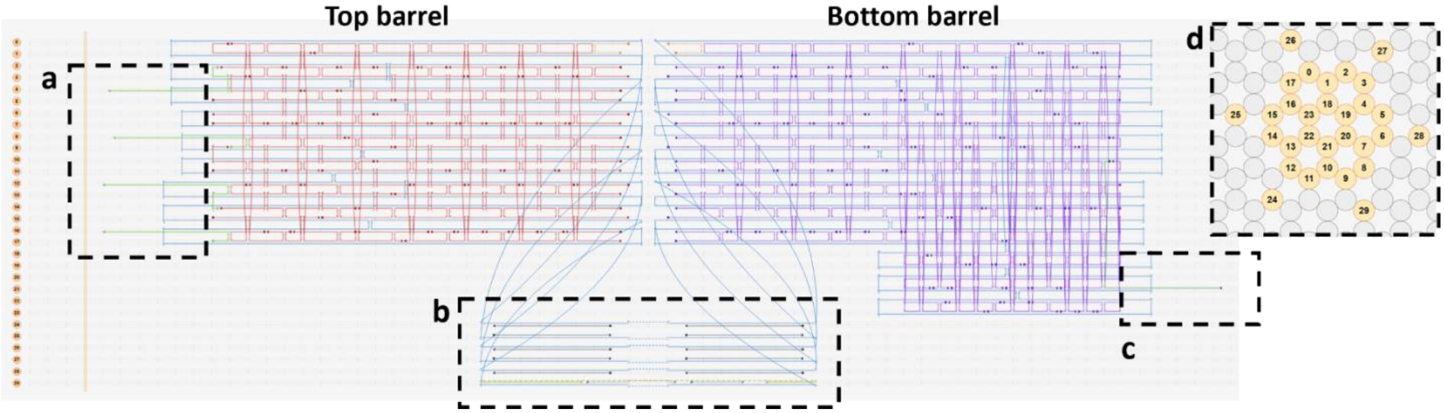

Supplementary Figure S1. caDNAno drawing of the DNA origami NanoDyn (ND): (**a**) 4, 24 nt ssDNA overhangs protruding from helixes 4, 8, 12, and 16 which anneal to a 25 nt ssDNA biotinylated oligonucleotide. There is a 22 bp overlap, leaving a total of 5 nt's unpaired spacers. (**b**) 6, 116 nt sections of ssDNA scaffold sequences attaching the top and bottom barrels of the ND referred to in the main text as 'loops". (**c**) 30 nt ssDNA overhang protruding from helix 21 of the ND for attachment to the DNA tether handle. (**d**) Cross-sectional view of the bottom barrel of the ND. The 6 helixes 24-29 indicate the routing of the loops outside the main body of the structure.

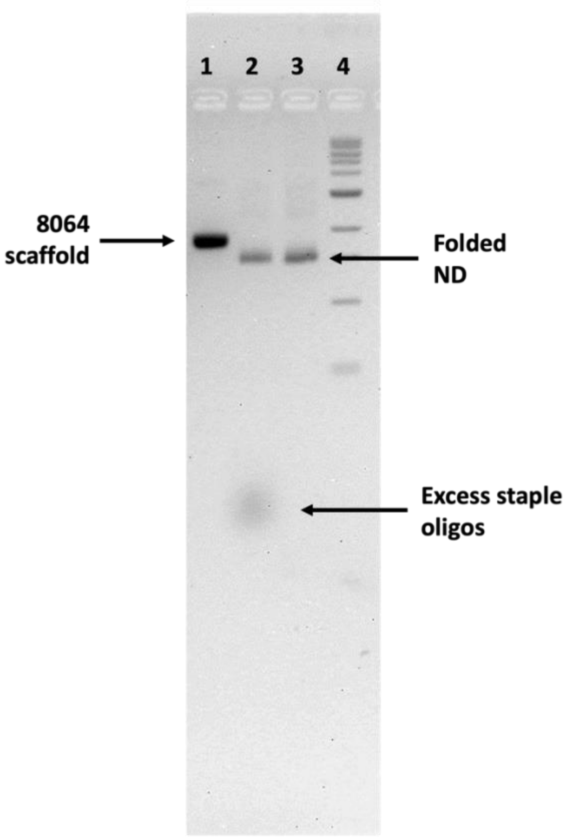

Supplementary Figure S2. Example of a NanoDyn fold and purification of the base version of the device (no zippers). **(1)** 8064 nt ssDNA scaffold **(2)** Unpurified ND omitting the L6 and L3 zippers. **(3)** PEG purified ND omitting the L6 and L3 zippers. **(4)** 1 kb DNA ladder.

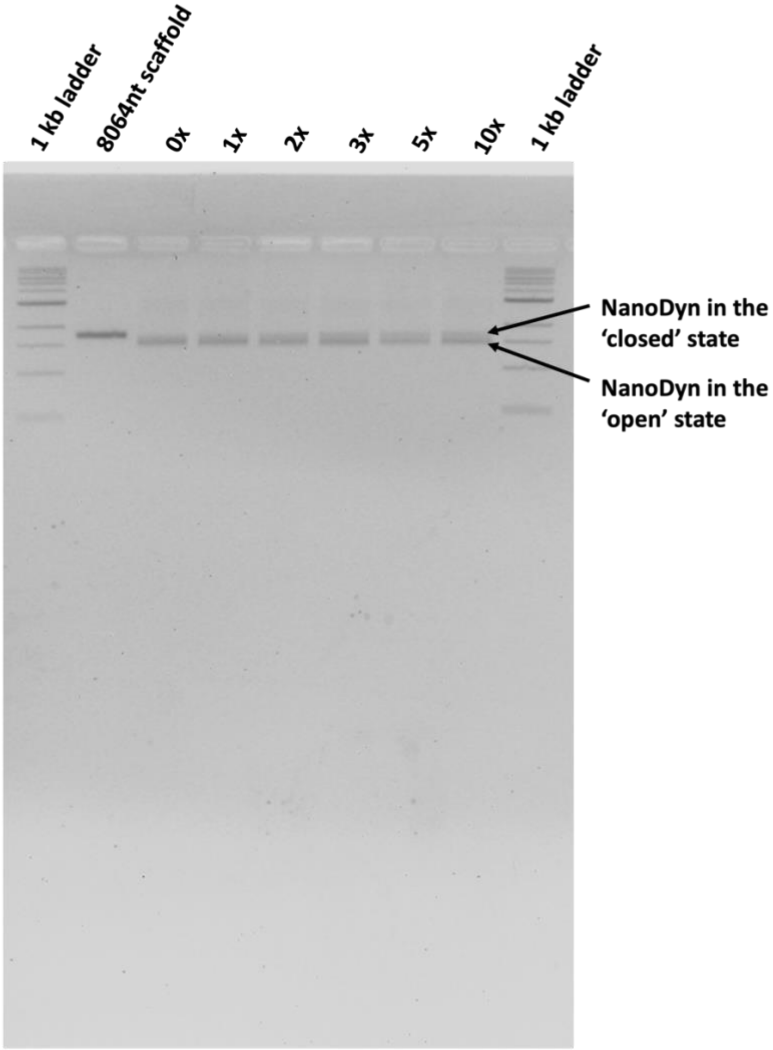

Supplementary Figure S3. Representative ND zipper incorporation gel for L6-17nt. From left to right, the L6-17nt zipper was titrated from 0x to 10x to determine the optimal zipper concentration. The lower band is ND that either has no zipper incorporated, or has a zipper, but in the course of electrophoresis has not remained closed. The upper band is ND that has the zipper incorporated and remained closed.

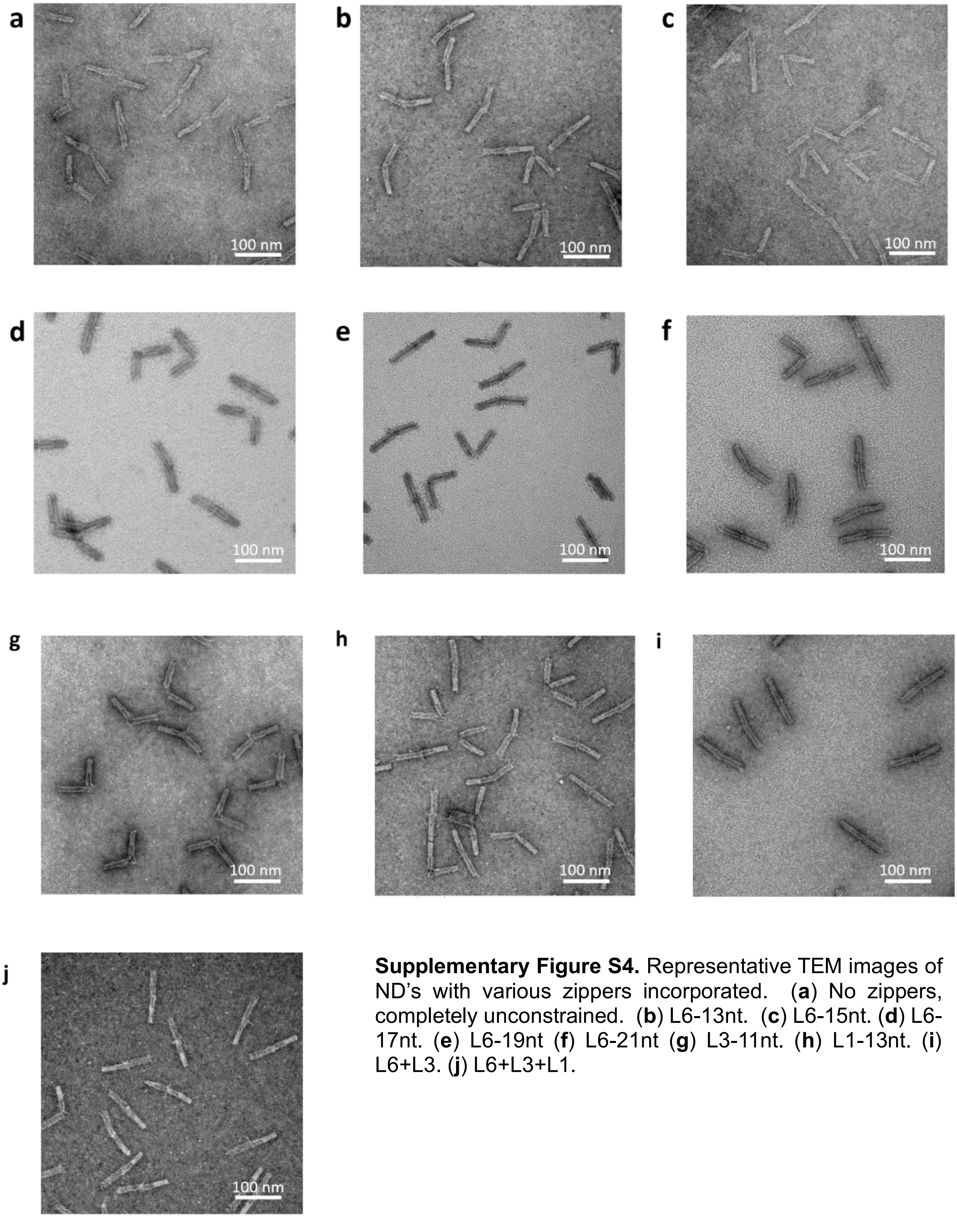

Supplementary Figure S4. Representative TEM images of ND's with various zippers incorporated. (**a**) No zippers, completely unconstrained. (**b**) L6-13nt. (**c**) L6-15nt. (**d**) L6-17nt. (**e**) L6-19nt (**f**) L6-21nt (**g**) L3-11nt. (**h**) L1-13nt. (**i**) L6+L3. (**j**) L6+L3+L1.

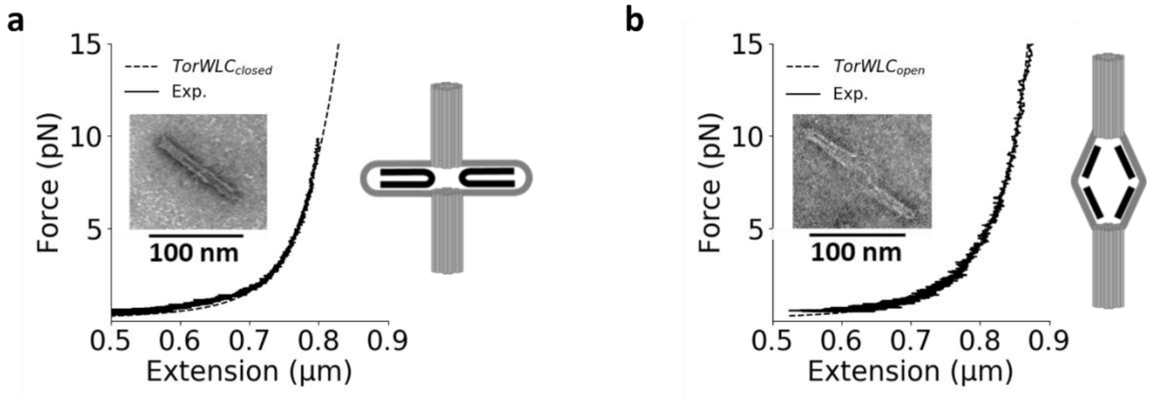

Supplementary Figure S5. Representative force extension curves for (a) a fully constrained closed NanoDyn, and (b) a fully open NanoDyn.

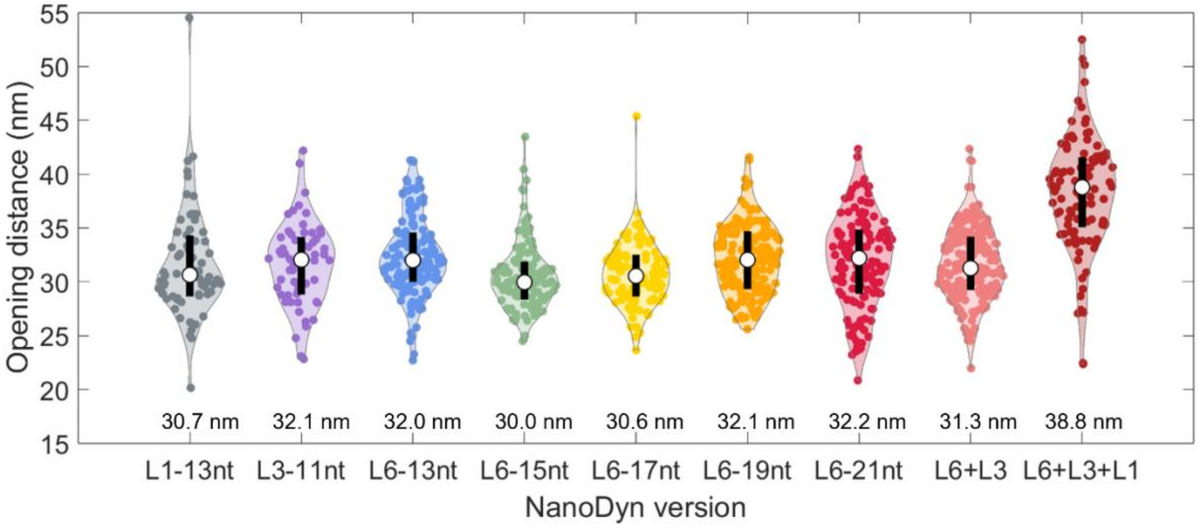

Supplementary Figure S6. The opening distance distribution for each ND version: Each colored dot represents the opening distance for each opening event. The white dot indicates the median opening distance which is listed below for each device version and the black bar is the first quartile above and below the median. The median opening distance, mean, SD, SEM, and interquartile values can be found in **Supplementary Table S2**. The L6-13nt + L3-11nt (pink) and L6-13nt + L3-11nt + L1-13nt (dark red) device names have been abbreviated L6+L3 and L6+L3+L1 respectively. The L6+L3+L1 distribution had 4 low distance outliers removed as the opening event occurred at the end of the data collection period and thus, an accurate opening distance could not be determined.

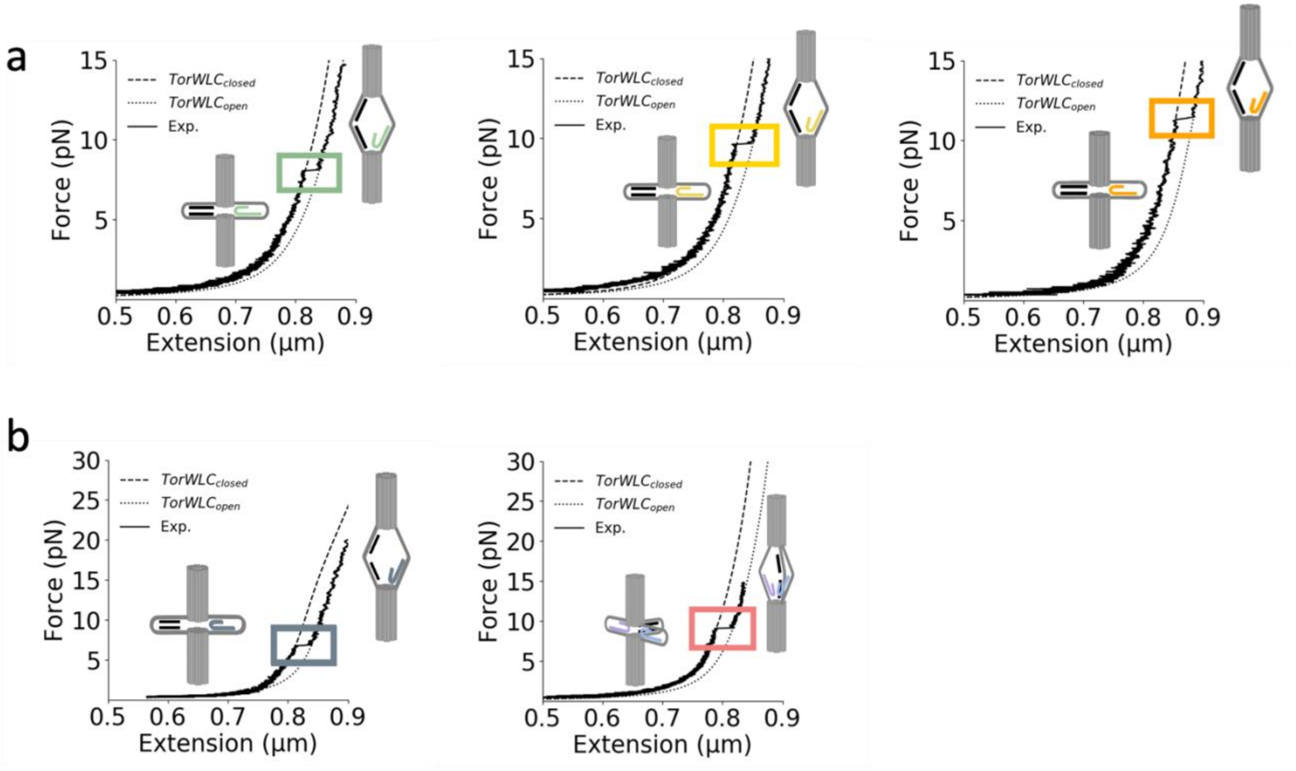

Supplementary Figure S7: Zippers of variable length and number modulate the opening force: Force extension data was fit to a Torsional spring + worm-like chain model (TorWLC) for both the closed ($TorWLC_{closed}$) and open ($TorWLC_{open}$) states. (**a**) Representative force extension curves for L6-15nt (green), L6-17nt (yellow) and L6-19nt (orange). (**b**) Representative force extension curves for L1-13nt (grey), L6+L3 (pink).

a

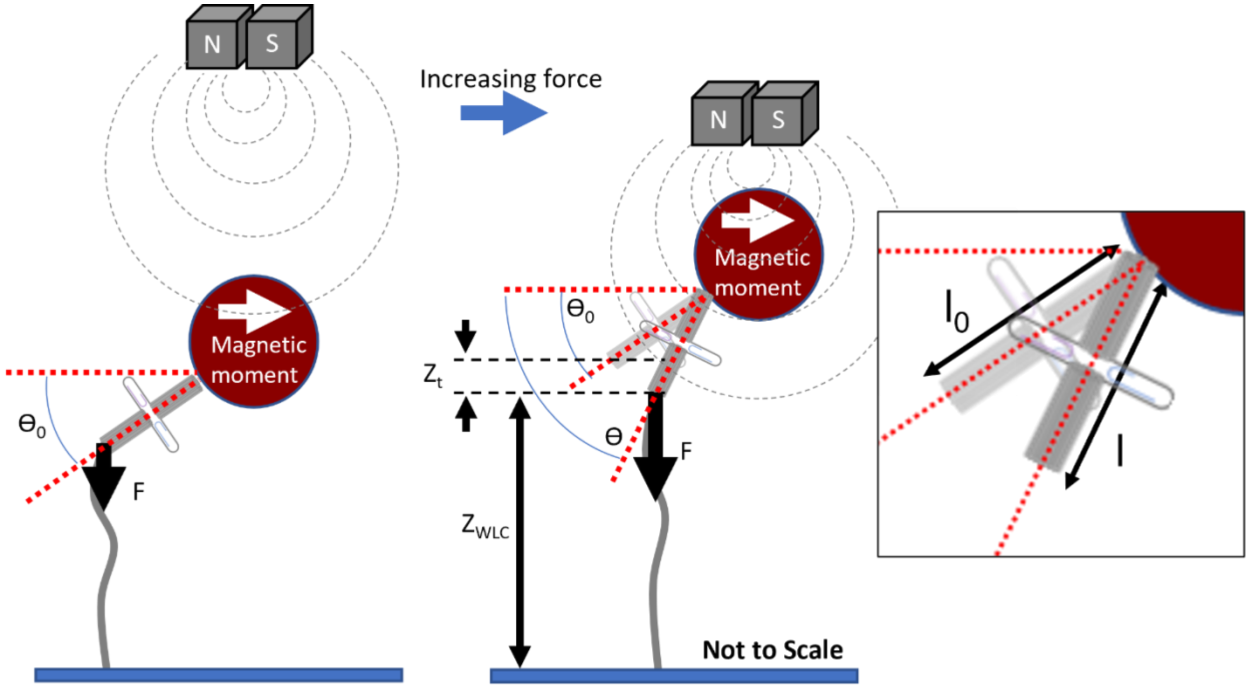

b

Color ID	Device version	K_θ (pNnm/rad)	Θ_0 (rad)
	L6-13nt	$2.19 \cdot 10^3$	-1.41
	L6-15nt	$1.93 \cdot 10^3$	-0.17
	L6-17nt	$2.12 \cdot 10^3$	-0.19
	L6-19nt	$3.72 \cdot 10^3$	-0.40
	L6-21nt	$1.97 \cdot 10^3$	-1.11
	L3-11nt	$1.97 \cdot 10^3$	0.15
	L1-13nt	$2.56 \cdot 10^3$	-1.18
	L6+L3	$4.34 \cdot 10^3$	-0.01
	L6+L3+L1	$4.38 \cdot 10^3$	-0.65
	Flow incorp: no zipper	$5.56 \cdot 10^2$	-0.60
	Flow incorp: L6-13nt	$2.33 \cdot 10^3$	-0.10
	Flow incorp: L6+L3	$2.55 \cdot 10^3$	0.14

Supplementary Figure S8. Torsional spring + Worm-Like Chain model (TorWLC): (**a**) This model was developed to account for variable torque applied to the ND based on the relative position to which the ND binds to the bead surface and the moment of the bead which will align with the applied magnetic field. The angle Θ will increase from its initial value Θ_0 until the force vector, F, is parallel with the lever arm (the NanoDyn). Z_{WLC} is the extension due to the Worm-Like Chain extension of the dsDNA tether and Z_t is the extension due to the change in the angle Θ of the NanoDyn relative to the horizontal. l_0 is the initial length of the NanoDyn and l is the final length of the NanoDyn. (**b**) The fit parameters listed were obtained for each of the force extension plots in the main body of the paper. K_θ is a torsional spring constant.

a

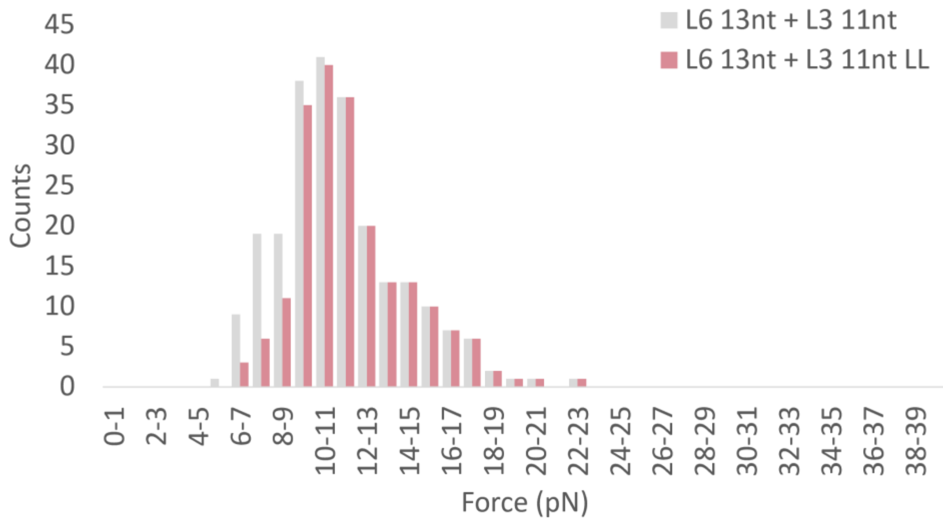

b

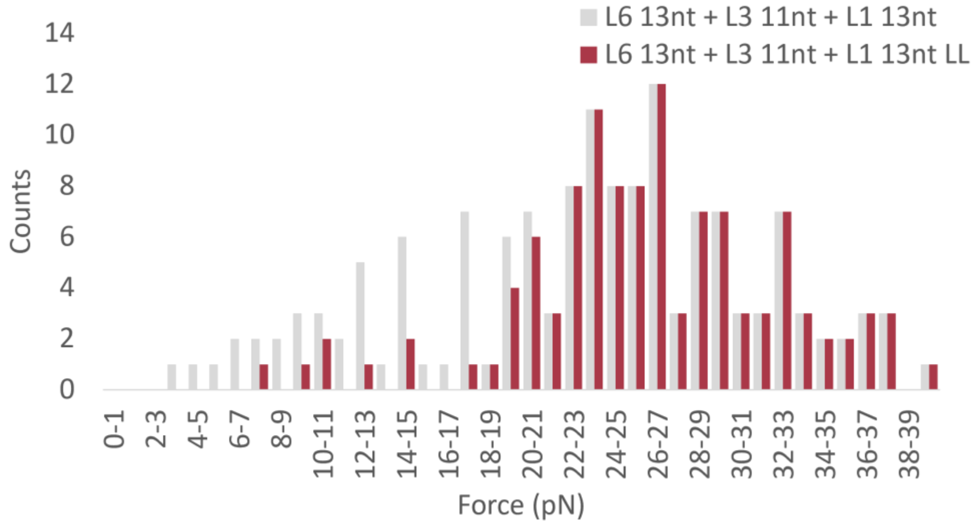

Supplementary Figure S9. Verification of zipper incorporation in the 2 and 3-zipper ND's using log-likelihood analysis. The data remaining after the log-likelihood analysis is denoted with "LL" in the legend. (**a**) Histogram of opening forces for the L6+L3 ND before (grey) and after (pink) log-likelihood cleanup. (**b**) Histogram of opening forces for the L6+L3+L1 ND before (grey) and after (dark red) log-likelihood cleanup.

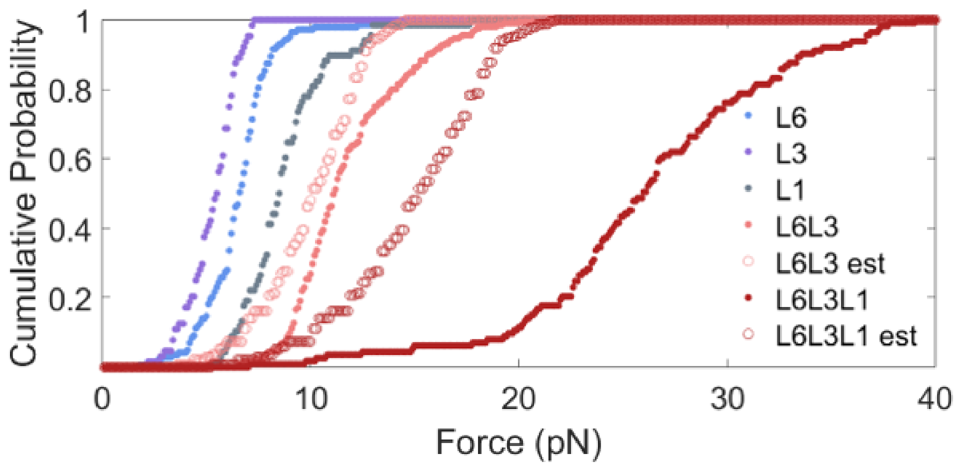

Supplementary Figure S10. Multi-zipper ND opening force estimation using cumulative sum addition. Experiment data are shown as solid dots and the estimates (est) as open circles. To estimate the L6+L3 opening force (pink) we summed the cumulative probability for the L6-13nt zipper (blue) and the L3-11nt zipper (purple). To estimate the L6+L3+L1 device (dark red) we summed the cumulative probability of L6-13nt zipper (blue), the L3-11nt zipper (purple), and the L1-13nt zipper (grey).

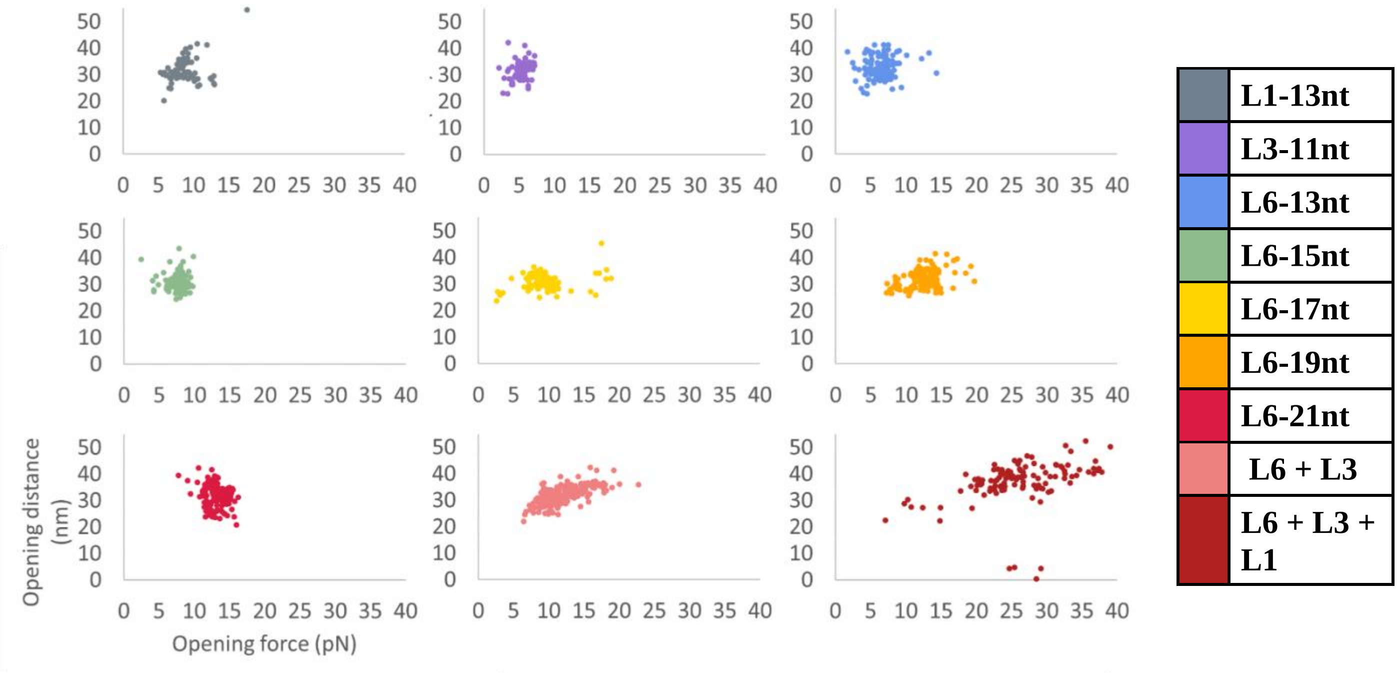

Supplementary Figure S11: For each opening event, the opening force vs opening distance was plotted for different ND versions. Note that the 4 low-force point in the L6+L3+L1 plot were the outliers removed from the distribution in **Supplemental Fig S5**.

a

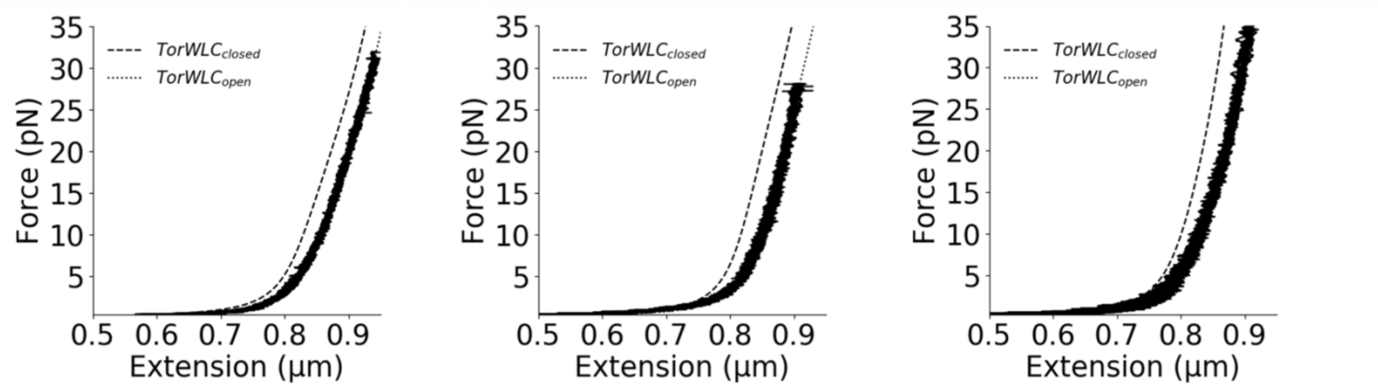

b

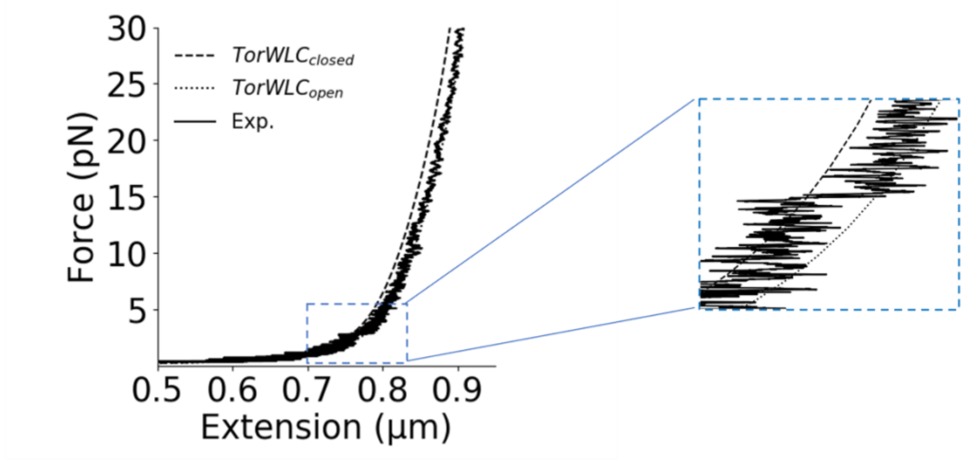

Supplementary Figure S12. Force extensions curves of the ND during unloading. (**a**) From left to right: 10 overlayed traces each of L1-13nt, L6-13nt, and L6+L3+L1, respectively. These unloading traces are the counterparts to the loading traces in main Figure 4. (**b**) Although relatively rare, closing events were occasionally observed. Most closures occurred at low force and are not easily distinguished in the bead fluctuation data. This event was collected from a L6+L3+L1, 3-zipper ND.

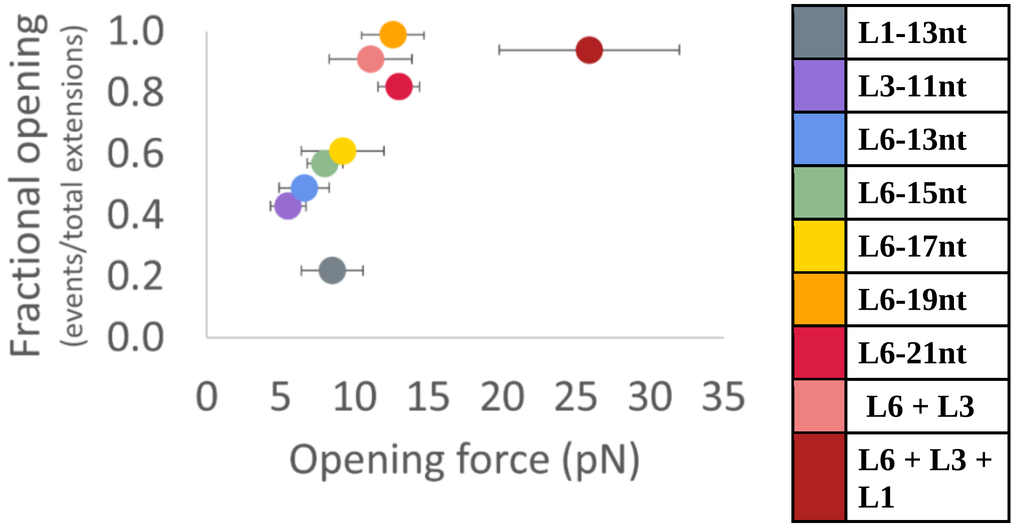

Supplementary Figure S13: Fractional opening of each ND version compared with its median opening force. The horizontal error bars are the standard deviation of the opening force. Numerical values can be found in **Supplementary Tables S1** and **S4.**

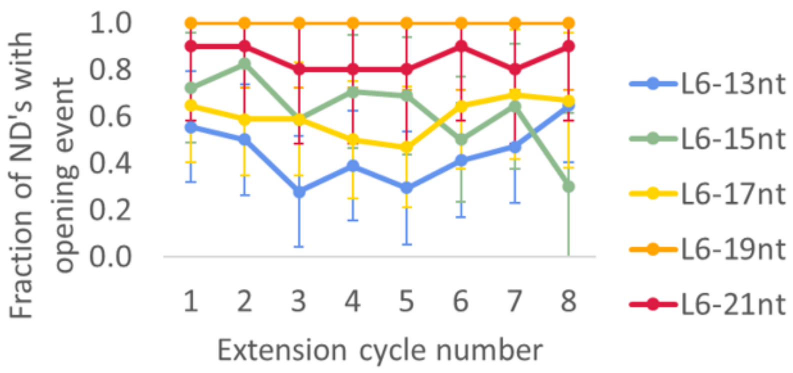

Supplementary Figure S14: Normalized fraction of ND openings per extension cycle. The standard error was calculated as 1/Sqrt(N) samples measured at the respective cycle.

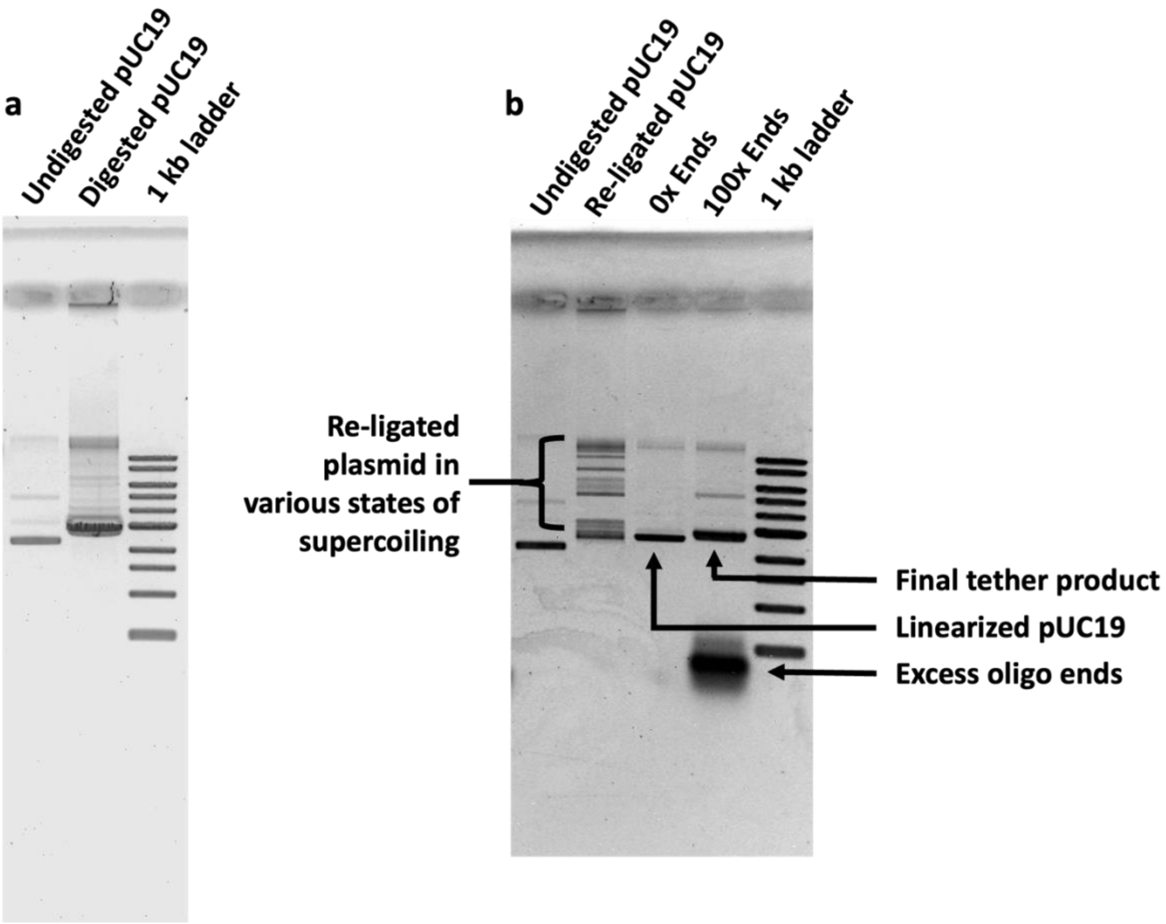

Supplementary Figure S15. Tether preparation for attaching the ND to the microscope slide. (**a**) Restriction enzyme BsaI was used to linearize a pUC19 plasmid. This leaves 4 nt sticky ends to which additional oligos are subsequently ligated. (**b**) 2 pairs of oligos are annealed to the linearized pUC19 sticky ends using T4 DNA ligase, resulting in a tether with a 30 nt overhang on one end and a 60 nt overhang on the other. We used 100x excess of the oligo ends for the final product, preventing recyclization and multimer formation of the pUC19 plasmid. This final product was then HPLC purified to remove excess ends (see **Methods**).

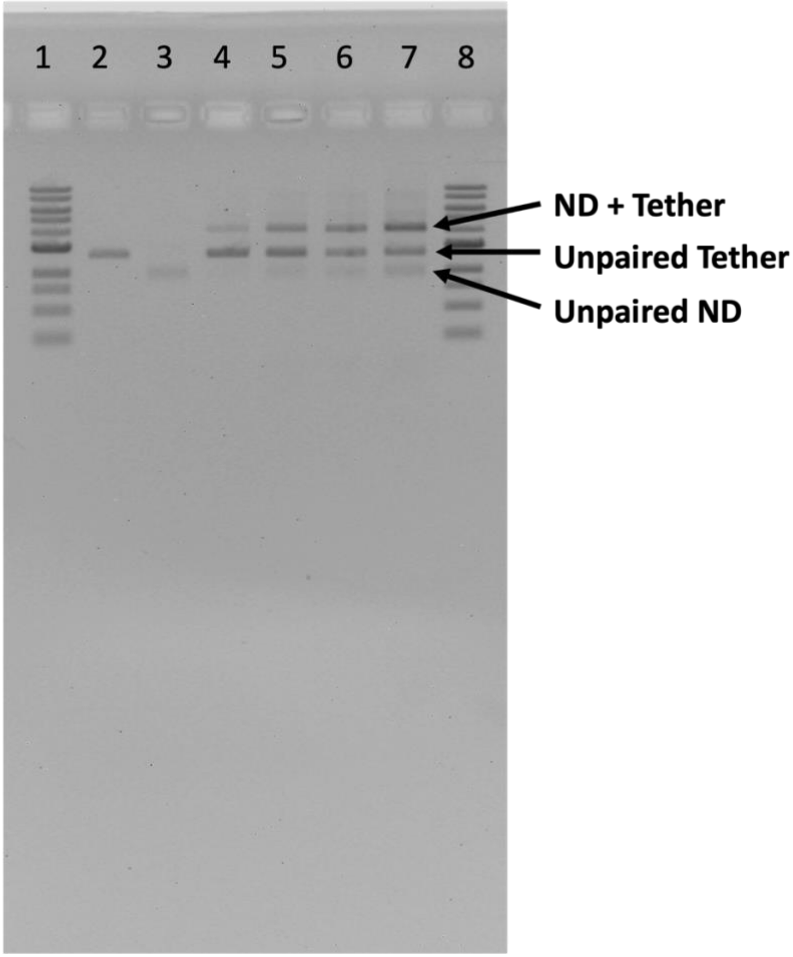

Supplementary Figure S16. Tether anneal to the ND: Tethers were annealed by titrating the amount of ND relative to the tether, which is held fixed. The ideal ratio maximizes attachment while minimizing free ND. From left to right: (**1**) 1 kb ladder. (**2**) dsDNA tether. (**3**) ND with L6-15nt zipper incorporated (**4**) 0.25x. (**5**) 0.5x. (**6**) 0.75x. (**7**) 1x. (**8**) 1 kb ladder.

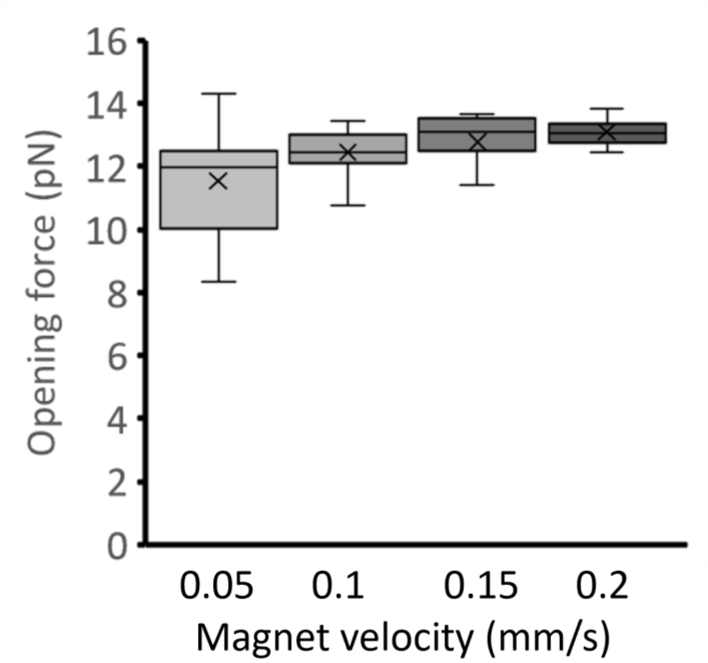

Supplementary Figure S17. Statistics of the opening force at different magnetic velocities: median (middle horizontal line), interquartile (top and bottom box borders), average (x) and standard deviation (uncertainty bars).

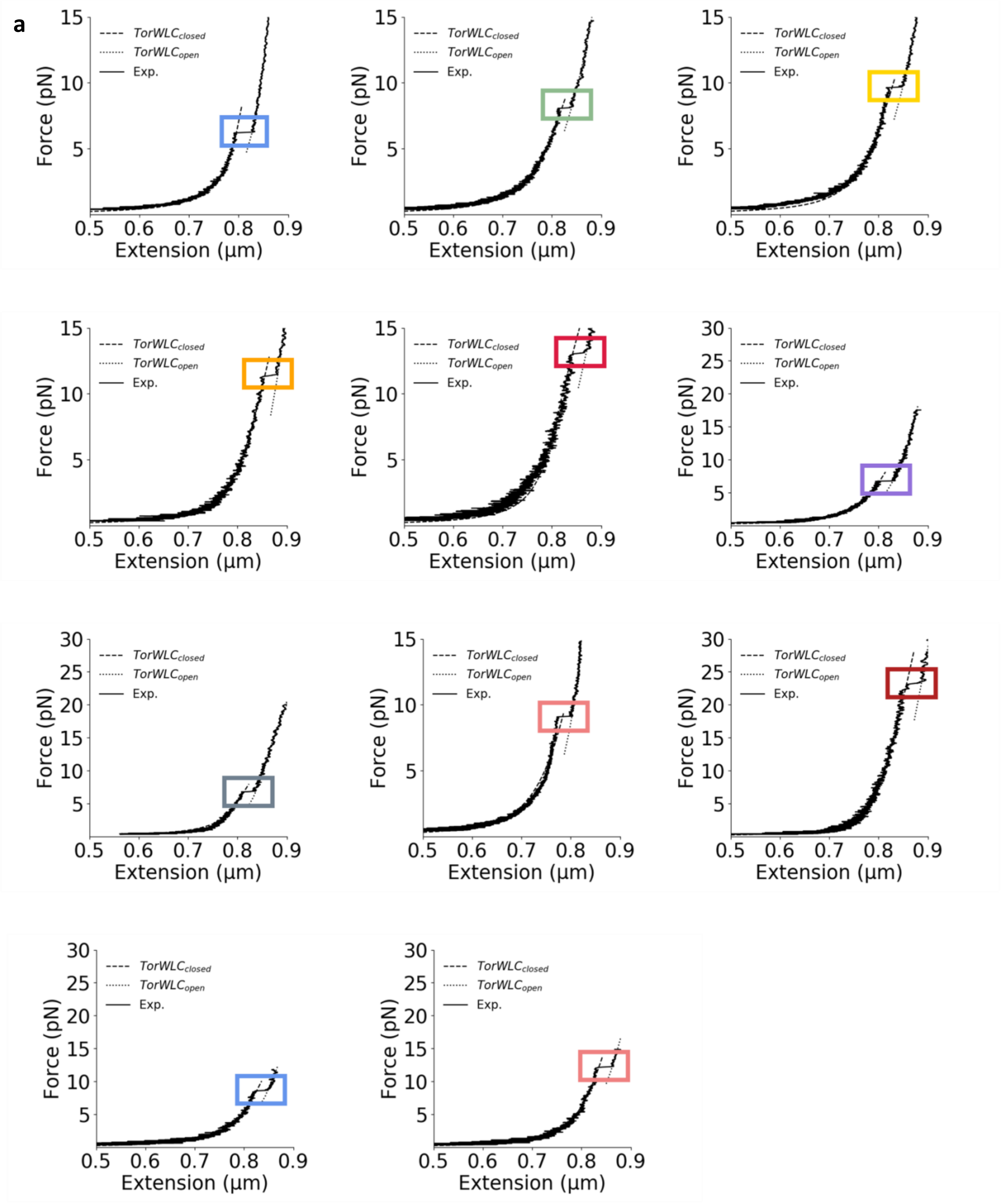
a
Force (pN)
Extension (μm)
TorWLCclosed
TorWLCopen
Exp.

b

Color ID	Device version	K_θ (pNnm/rad)	Θ_0 (rad)	ΔL (nm)	l_0 (nm)
	L6-13nt	$6.23 \cdot 10^3$	-0.08	34.0	90.9
	L6-15nt	$1.76 \cdot 10^3$	-0.55	35.0	82.9
	L6-17nt	$1.96 \cdot 10^3$	-0.53	36.7	84.8
	L6-19nt	$2.48 \cdot 10^3$	-0.72	46.9	73.9
	L6-21nt	$2.03 \cdot 10^3$	-1.11	34.0	94.5
	L3-11nt	$2.08 \cdot 10^3$	-0.31	44.5	72.6
	L1-13nt	$1.35 \cdot 10^3$	-1.26	34.0	66.3
	L6+L3	$1.84 \cdot 10^3$	-0.09	52.7	53.2
	L6+L3+L1	$4.47 \cdot 10^3$	-0.74	43.2	87.0
	Flow incorp: L6-13nt	$2.30 \cdot 10^3$	-0.29	34.0	85.2
	Flow incorp: L6+L3	$3.35 \cdot 10^3$	-0.32	34.0	91.5

Supplementary Figure S18. Results of TorWLC model with change in effective device length at rupture point. (**a**) Representative force-extension curves for all devices with rupture events shown in the main body of the paper, but fit with a TorWLC model that allows the effective device length after rupture and the effective tether length added during rupture as additional fit parameters. (**b**) The fit parameters for each of the fits shown in (**a**).

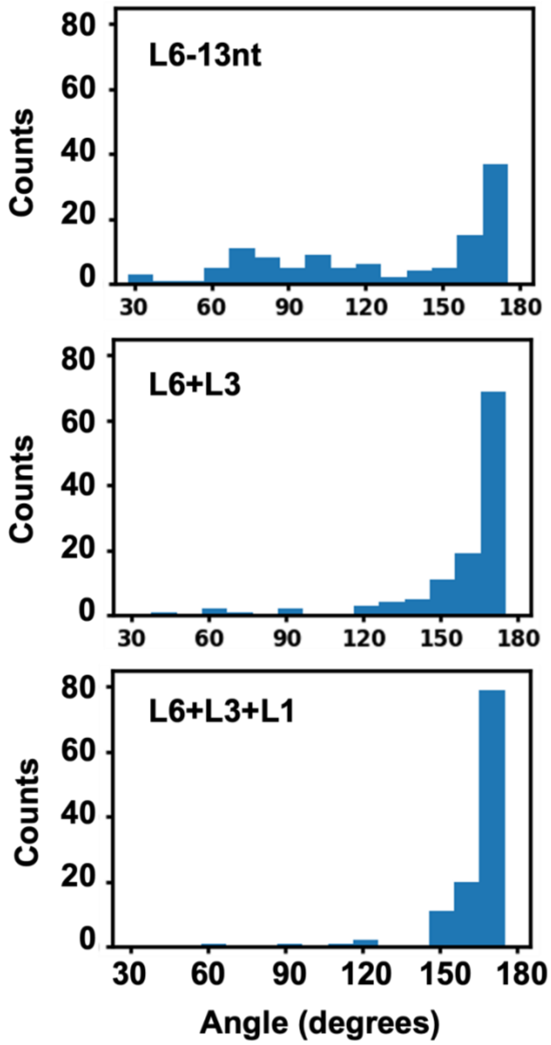

Supplementary Figure S19. Angle between the two barrels of the ND as determined from TEM images for different zipper configurations (L6-13nt, L6+L3, L6+L3+L1). 180 degrees is for configurations where the barrels are parallel. The ND devices were deposited in the buffer that was used for the MT measurements. As more zippers are included the angle distribution is reduced. However, the variation in angle prevents a well-defined measurement of the gap size.

Color ID	NanoDyn version	Median opening force (pN)	Mean opening force ± SD (pN)	SEM (pN)	Interquartile (pN)	Total opening events measured	Total # of molecules studied
	L1-13nt	8.5	8.7 ± 2.1	0.3	1.9	68	13
	L3-11nt	5.5	5.3 ± 1.2	0.1	1.6	64	11
	L6-13nt	6.6	6.5 ± 1.7	0.1	1.6	143	18
	L6-15nt	8.0	7.7 ± 1.2	0.1	1.1	113	11
	L6-17nt	9.2	9.5 ± 2.8	0.3	2.0	108	17
	L6-19nt	12.6	12.5 ± 2.1	0.2	2.5	162	13
	L6-21nt	13.0	13.1 ± 1.4	0.1	1.6	136	10
	L6 + L3	11.1	11.6 ± 2.8	0.2	3.3	205	23
	L6 + L3 + L1	25.9	26.0 ± 6.1	0.6	7.0	113	11

Supplementary Table S1: Table of measured forces, the associated error, and event counts for different ND versions.

Color ID	NanoDyn version	Median opening distance (nm)	Mean opening distance ± SD (nm)	SEM (nm)	Interquartile: (nm)
	L1-13nt	30.7	31.7 ± 4.9	0.6	5.0
	L3-11nt	32.1	31.7 ± 3.7	0.5	4.8
	L6-13nt	32.0	32.4 ± 3.6	0.3	4.1
	L6-15nt	30.0	30.6 ± 3.2	0.3	3.0
	L6-17nt	30.6	30.7 ± 2.9	0.3	3.4
	L6-19nt	32.1	32.2 ± 3.2	0.3	4.8
	L6-21nt	32.2	31.9 ± 4.2	0.4	5.4
	L6 + L3	31.3	31.6 ± 3.3	0.2	4.4
	L6 + L3 + L1	38.8	38.2 ± 5.2	0.5	6.0

Supplementary Table S2: Table of measured opening distances and the associated error for different ND versions.

Color ID	Zipper name	Force-sensing strand anchor sequence + 5 nt poly-T spacer (5'-3')	Zipper binding sequence (5'-3') (*end connected to poly-T spacer)	Zipper melting temperature (°C)
	L6-13nt	TTTTTTCTGCGAACGAGTAGATTTAGTTTGACCAT	ATCAATTCTACTA*	36.4
	L6-15nt	TTTTTTCTGCGAACGAGTAGATTTAGTTTGACCAT	GCATCAATTCTACTA*	46.0
	L6-17nt	TTTTTTCTGCGAACGAGTAGATTTAGTTTGACCAT	TGGCATCAATTCTACTA*	52.6
	L6-19nt	TTTTTTCTGCGAACGAGTAGATTTAGTTTGACCAT	GGTGGCATCAATTCTACTA*	57.2
	L6-21nt	TTTTTTCTGCGAACGAGTAGATTTAGTTTGACCAT	AAGGTGGCATCAATTCTACTA*	59.8
	L3-11nt	CAAGAACGGGTATTAAACCAAGTACCGCTTTTT	*TAAGTCCTGAA	35.1
	L1-13nt	ATTAGACTTTACAAACAATTCGACAACTCGTATTTTTTT	*GGTTATCTAAAAT	36.0

Supplementary Table S3. Table of the individual zipper designs used in the main text. The anchor sequence was designed such that the melting temperature was >60 °C. The 5nt poly-T (underlined) was added so that there would not be a kink in the zipper oligo where the binding and anchor region meet. Melting temperatures were calculated using the IDT Oligo Analyzer Tool, using the experimental salt conditions of 100 mM NaCl and 10 mM $MgCl_2$. The oligo concentration was set to 40nM, which is the concentration used during post-fold incorporation of the zippers.

Color ID	NanoDyn version	Total opening events / total extension cycles		Median opening fraction	Mean opening fraction of individual NDs ± SD	SEM	Interquartile:
	L1-13nt	68/304	22%	0.13	0.28 ± 0.31	0.09	0.17
	L3-11nt	64/148	43%	0.33	0.47 ± 0.29	0.09	0.31
	L6-13nt	143/292	49%	0.43	0.46 ± 0.23	0.05	0.33
	L6-15nt	113/199	57%	0.59	0.66 ± 0.27	0.06	0.52
	L6-17nt	108/178	61%	0.60	0.56 ± 0.26	0.06	0.35
	L6-19nt	162/164	99%	1.00	0.99 ± 0.04	0.01	0.00
	L6-21nt	136/166	82%	1.00	0.83 ± 0.27	0.09	0.33
	L6 + L3	205/225	91%	1.00	0.92 ± 0.12	0.03	0.10
	L6 + L3 + L1	113/120	94%	1.00	0.95 ± 0.09	0.03	0.03

Supplementary Table S4: Table of opening fraction characteristics and the associated error for different ND versions.

Supplementary Table S5. Table of oligonucleotides used to fold the base NanoDyn structure.

Name	Sequence
oligo1	ATGCGTTCTAGCTGATAAACAGGAAATCCTGGAGGCCGCTAT
oligo2	TAATGAGAGATCTACAATCAATCAAAAGAGTAACTATC
oligo3	TTTCAGACAACACCAGTAATGGATCATCAGAGCTGGTCTGGTCAGTCCG
oligo4	CCTAAAGCGTTAAAAGGTTTTGACGTCACTGCTTGTAGAACGTCATTTC
oligo5	AGTGTTGGAACGCGTTAGTATCGCTCAAGAACGCGCCTGTTTATCAACA
oligo6	GAATAGCAATATATTAATTACTGAGAATACAACATGTTCAGCGGGTAAA
oligo7	AAAATCCTCTTCTGTAAGAATACGCCAACTGTCCAGACGACGTCATAAA
oligo8	GATGGTGGTTTGAAATACCCAGTCGGGACGAAAATTTTGGGGGCAA
oligo9	TGGAGCACTAGTGCCACGCCGGAGAGGGTAGGATAATCAATC
oligo10	GCGGTCCTAATGAAACTCACAACCGAGCGTTCTTCGCGTCCGCTGC
oligo11	TCAGAACTGAAAAACAGCAGGTCATTGCCTGGGTAATCAATT
oligo12	CTGATTGGGGCGCCAAGCATAGTGAAATTTTTCACGGTCATAGCGC
oligo13	AATCGCAAGACAAATTCCAGTTTGG
oligo14	TTTTTCACCGAGATAGGGCGATGGTCAGTAAAGATTCAAAAGGAAGCCT
oligo15	AATTTCACTTATAAGGGCGATATCAAACAAGGCCGGAGACAGCCCTGTA
oligo16	TTTAATGGTTCCGAACCATCATCACCTTACCATCAATATGATGTTGTAC
oligo17	TGTCTCACTGATCAGTTGAGGATCCCCGGGTTTAATTGGGCG
oligo18	TCGACCGTGTGATAAGAGGCACCGACAA
oligo19	GCCATGGTCATTTCTGCCAGCACGCCCCCTGTATTTACTCTGCTG
oligo20	TATAAAGCCAACATATGCGTTA
oligo21	GCCAGTACCCGCTTGGGAAGAAAGCCGGACGCCAGAGATTGT
oligo22	AAGGTAAGTGTGTTCAGCAAACGCAACCAGCTTACACCGATA
oligo23	CCTCAAAGAGAATACGGCATCCCGCCGCGCGCGTAATTAAAGGGCG
oligo24	GGGTAGCTGTTTCCTGTAAGTGTACCAGTAAAAGAATATCAC
oligo25	TCACTGTTGCCCTGCGGCCAATCCGCCGGGCGCGGT
oligo26	AATTAATGCACAGTAGGGCTTAATTAGAAAAAGCCTGTAGAAAAC
oligo27	GGTTTCTCATTGCAGGCGCTTTTATCAGTCGCTGAAACGAGGCATCTTT
oligo28	TCGACAATAACGCCATATTTAACAAAACACCGGAATCATTTAGTT
oligo29	CATCCCTGGTGTCCAGCATCAGAATTTCACGCATAAGAGGCTAAAAGAA
oligo30	CTGAGTAATTCATGTAATTTAGGCAATAAGGCGTTAAAACCTAAA
oligo31	AGCCGCTGCGACGAGCACGGGAGCCGCTATTGTCGGATTCTCCGTCGTT
oligo32	AGTGCTCAATAATATTAAGTAGAAGATTAAGAGCCAGC
oligo33	AACGCCGGTGCGTGCCTTCGAATTCGTAATCTAATGAGGCAG
oligo34	GGACGCGCCTCGGGCCGTGTTATC
oligo35	TCCAAAAGGAGCCTTAAAGGCCGCT
oligo36	GTTGCACAGGCGGCCATCCCATCGTTAATAAAGTATTTTCGA
oligo37	AGTCCGTAAAATTAAACGGGTACAATCGGCGGACATAATGCT
oligo38	ACGGTACATCAAACGTAATAACCTCACCGGAGCCTCTTTAAA
oligo39	GCACGCCAGCCCAGTCCGTGGAGCCGCCACGCTGGCGACGTT
oligo40	GTCCTTAAATGATAGACGCCAGTGCCAAGCTGCAAGGCGAACTCACTGT
oligo41	CCTCAGCAGCGATACCAAGCGCGAA

oligo42	GCATCGGGGCTTGCAGGGAGTTTAATTGTATCGGTTCGCACTTGGT
oligo43	CGGCTACACCGATATATTCGGCTTGCTTTCGAGGTGCGGGGTTTGC
oligo44	CTAAAGAAACAACCATCGCCCTTAAACAGCTTGATGGCTGGATACA
oligo45	AGTTTCCAAAAGCCGCGCCGACAATGACCTTTTTCCCAACCT
oligo46	GACCCCCGGCACCGCTTCTGGATCTGCCAGTTTGAATCATACAACG
oligo47	TACACTAAACCAGGCAAAGCGATGGGCGCATCGTAATTAGCATGCG
oligo48	AAAACGACCATTCAGGCTGCGGGATAGGTCACGTTAGCCTCAATTA
oligo49	GTTGTAAACGCCAGACATCACCGTTGTATGACCTGAAAACATGCAACAG
oligo50	CACTCCAGCCAACGACAGTATC
oligo51	ATCAACGGCGGATTGACATCAAATATTTAAA
oligo52	CCCACGCCAGGGAACGGCAGCGCCATGTTTAAGTTGGGCGTG
oligo53	TTAATGTGCTTTCAGAGCGGAATTTGTGAGATTCTGCTCCAG
oligo54	TTTCATCCAGCTCATAGCATGAGGCTATAGGTGAGGCGGTCAAGTC
oligo55	TGTTTGGGTAAACGACGTTTCTCCGTGGTGAAACA
oligo56	TCTGGCCGGAACGCGATGAACAGAGTCTCACCAGCAGAAGATTAGC
oligo57	AGTAGCATTAACATCCAATAAGGGGACGGCTTTCCAGCGATTAAAGACA
oligo58	TTGTAAAGGGAACAGGTGCGGAAAAATACGTAATGCCACTACTGGGAAG
oligo59	AATCCCCAAAAATTAATGCTGAGAGCCAGCATCGAGGTACGG
oligo60	CAAGTGTAGGTTGGCAAATCAACAGACTCCAACGTCAAAGGGTTG
oligo61	TTATTTCAGGCAAGGCAAAGAACCGTGCTGCCGGAAAACACTGTAGCAA
oligo62	GGAGGTGAGACCTCAATCAATATCAAAACCGTCTATCAATCAAAA
oligo63	ATACTTTAAATTAAGCAATAAGGTGTAGCCATTCGAAGAGGCTTGAGGA
oligo64	TGATCAAATCGCTGAACCTCAAATGGCCCACTACGTGAAATCGGC
oligo65	CAAAAACGAGCATAAAGCTAACGTAATGCAACTGTGAAGGCAATGAGGA
oligo66	ATAAGCGATTCAACAAAAATCTAAAGCACCCAAATCAAGTTTCCTGTTT
oligo67	ATGCAATGCCTGAGTAATGGATAAAAATTTTTAGAA
oligo68	TTTCACCGCCTGCAACAAATCGGAACCCTATGCAGCAA
oligo69	GGAAAGCGAAACCACACAGATGCCACCGTCGGTGGTGCCTTT
oligo70	CTACGATTTATTTTTATAGAAAAGATTTTGTTAAAATTACAA
oligo71	GGCAATGTGAGCTATCGGCCAACGCGCGGAGAGTTGGCTATTGTAT
oligo72	ATTTATTTTTCCGAGTATATGTACAATTTTTGTTAAATAACA
oligo73	ACCTACAGACATTCGAGCCGGAGGGTGG
oligo74	GGTAAAAATCGATGAAGGGTAAAGCGGCAGCGGGTTACTGAGCCT
oligo75	TTGCGTAACCAGGAGCGCGTTGCGCGTGCCAGCTGCATACGC
oligo76	CTTATGTGTAGCGGTCACAGCGGTCGCAAGAATGCCAATTAA
oligo77	GTAATATCATTTGCAACGCGGTCCGTTTGCGTGGTCGATCCACCGG
oligo78	AACCGTCTGACACACGAAAGCCTGGCGGTTTGCGTATTCCCT
oligo79	CAGAGAAGTGGAGCTTGGCCGTAATTTGCCCCAGCAGGAACC
oligo80	AGAGAGGCCAGAAAAGGGAGCCCCGGGCGCT
oligo81	GGCCTCTAAGGGGGAATGTGAGCGAGTACGCATTACCCGGTTCTAT
oligo82	AGTATCAGAGCGTATAACGGTTGTATGCTGATTGCCGTCAGC
oligo83	CCACGTGGCAAATGCGCCCGCCTGGCCCTGAGGGAGAGGGGT
oligo84	GCAGTAAAACTTTTTTAACCAATATTCC
oligo85	AACTTGCCTGCCGCCAGGATCAAATCGTCGCTGGCAGCTGCC

oligo86	TGAATAATTTGCACGTAAAACAAGCGCATTAG
oligo87	TCACATCAATCAGTTCAGAAAACGCGAGAGGCTTTTGCAACCCTC
oligo88	ACATAAATCAGAGAAATAGCAGAAACGCAACATATAAAAGAAATATGGT
oligo89	GCAGCCTATTGAGTTAAGCACGATTTTTTGTTTACCCAATCCTAAGACT
oligo90	CAAAAATTGAATATGATTATCCCATAAATCAAAAACGGATGGCTAAACC
oligo91	AATTTTTACACGGAATTTTTACCCTGACTATTTTGATA
oligo92	TTACCTTGTGAGTGTAAACAGAAGAACGATATAGAAGGCTTAATATTGA
oligo93	CATTTAATGTAAATCCTAATTGAACCTCGCCCAATAGCAAGCTAAAGGT
oligo94	TCAAGAAAATTTTCCTAACGAGTTTTGACATCGTAGGAATCAACCGACT
oligo95	GCAAAAGAAGAAAAACATAGCGATA
oligo96	GCTAATAAACAGGGAGAAATATTGGATTATCCCCCTCAAATGGTTTTGC
oligo97	TTGCTTCCAATTTCAACGGATCAAAGAAAGAAGCAAAGCGGAAGAGTAC
oligo98	ATTAATTAACAAAATGAATACTAACATTAAAAAGATTAAGAGACTCCAA
oligo99	ATCCTTGTGATGAAGCGCAGATATTAATGAAAGACTTCAAATATCG
oligo100	ACAATGAGATAACCCACAAGATTACAGAATTT
oligo101	AATAAGAACCCAAAAAATACATACATAAGACAAAA
oligo102	AATTTTATCCTATTAGTTGCTA
oligo103	AGTTACCAGAAGGAAACCGAGATAGCTATCTTACCATTGAGC
oligo104	CCTTACGGTATTCTCCATATTATTTATCATAAATCAATATATTTTT
oligo105	TTAGCAACGATTGAGGGAGGGCAGACTGTAGCGCGCATTTCGTCACCAG
oligo106	TCGAGAACAAGCAAGCCGTTTATTAGAGCCAGCAAAATC
oligo107	TCAATAGAAAATTCACGCAAAGACACCA
oligo108	TTACCAGCCTTATTAGCGTTTCTCCCTCCGCCGCCACAAATAGTAAGCG
oligo109	AAAAGGTGGCAATAATAACGGAATGCAAGAA
oligo110	GGGCGACCGGCATTTTCGGTCCAGAACCCACCAGACCAGAAT
oligo111	CGGAAATAATCAAGTTTGCCTTACAACGAGGAACCTTGCTCAACCGGAT
oligo112	CATAAATCAGCGAGGCGTTTTAGCTGCCAGTTACAAAAAATAACC
oligo113	GAATTATCACCGTAATCAGTACAGACAGTTCAGGGAGTGCCGTCAAGAG
oligo114	GTCTTACCGCCCGACTTGCGGGAGGCGTCTTTCCAGAGCGTCGCT
oligo115	TGAGCCACAATGAAACCATCGGTAACGACTCAGAGATAAGTAGCATAGG
oligo116	GGATTATTTTAGCCTTAAATCAAGGAATCTTACCAACGCCTTAGA
oligo117	CATATTCAACACGTAGAAGAACTGGCATGATAAATAAGAACCAAT
oligo118	CCATTAGCAAGGCCGGAAGTCTTTCGCCACCCTGTATCATGAACGG
oligo119	TACAAACTTAGCGTAAGGTAATCTTACGCAGTATG
oligo120	CTGAGCCAGGATTATAAGAGGCTGAGACCCTATTTCGGAACCATAAAAC
oligo121	CCCTCAGAACCCAGACGTTAGTAAA
oligo122	CTTGATATTCACAAAGCATTGACAGGAGACCACCG
oligo123	ATTAAAGACCACCACCAGAGCAGAGCCGCCACCCTATAGCCCCGCC
oligo124	GGAAACTCAAGAGAACCCTCAGAGCCGCGCCACCCTCAGAGCTTTT
oligo125	GCGGATAATAGCAAGCCCAATCCTGTAGCATTCCAGCGACAGTATT
oligo126	GGTTGATCCACCACCCTCATTCCCTCATAGTTAGCATAGCAGCACC
oligo127	GAATAGGTCAGAACCGCCACCTCTAAAGTTTTGTCACGTCACTTTG
oligo128	TCATACAGCCTTGAGTAACAGTTCATCAGTTGAGATTTA
oligo129	AATTTACATAAACAGTTAATGACATTATTACAGGTCTATCATAAAA

oligo130	ATACATGAAAGTATGGATTAGCGGGGTTCATGTACCGTAACA
oligo131	TAATCTTCGTAACAAAGCTGCTCATTATACCAGTCGCTCAACTGCT
oligo132	CTGGCTGGTGAATAAGGCTTGTATGCGATTTTAAGCAACTAAGGAT
oligo133	TGTACAGGAGAAACACCAGAATTTAATCATTGTGAAAGTTTCGAAG
oligo134	TGAAAGAGGACAGACCGTACTCAGG
oligo135	CTGAATCATTACCCTGGGAAGAAAAATCATTGCTGATTTTTGTCAG
oligo136	GAACTAAAGACGACGATAATAGAGCTTATACGTTATATTATT
oligo137	TTAATTTCAACCGAGTAGTAAA
oligo138	GGAATTACGAGGCAGGTAATATCATTGAATACTTC
oligo139	GTTTACCCGGAACACCCCCTGTCGCGCAGTCTCTG
oligo140	CAGAGGGTAGTAAGAGCAACAAGAAAGATGCCCGTCGTTCCAAATCCTC
oligo141	GAACTTTAAAATAATCCTGATTGTAAGAAATTGCGTAGGAGAATA
oligo142	AAAATAGAGAATGAAGATGATGGCAATTGGTTTAACGTCAGAGAAAATA
oligo143	AGAGGTCAATATAATGCTGTAAGGACGTAAATCAAGACAAGAGTACCAG
oligo144	CCTTATAGTCACCACCAGAAGGAGTCGGGAGAAACAATATTTGAA
oligo145	CTTTAATATGTTTTAAATATGAACTGGCTCATTCAACCTTCATCGAGAG
oligo146	TAGTTGCATCATCATTTTGCGGAATCGCCTGATTGCTTTTAATTA
oligo147	CAGGTCAAGTACGGTGTCTGGATTACCTCCCTGACACCAGGCTAGCCCG
oligo148	CAAGAAGCCCTTTAAAAGTTTGAGCAAGTTACAAAATCACAAACA
oligo149	TCAAAGCGAACCAGACCGATTCCATATAACAGTTGATTC
oligo150	GTCATCATCATATTCCTACAGTAACAGTACCGGAAACAGTAACGT
oligo151	CTTTGCCCGAACGTGGCGAATTATT
oligo152	TAAAGAACGTGGTTGAAAGGAATTG
oligo154	TTTGAGGATTTAGAAGTATTAGACTTTACAAACAATTCGACAACTC
oligo155	TCTAAAATATCTTTAGGAGCACTAACAACTAATAGATTAGAGCCGT
oligo156	AGACGCTGAGAAGAGTCAATAGTGAATTTATCAAAATCATAGGTCT
oligo157	TTAACCTCCGGCTTAGGTTGGGTTATATAACTATATGTAAATGCTG
oligo158	TCGGCTGTCTTTCCTTATCATTCCAAGAACGGGTATTAAACCAAGT
oligo159	CCTGAACAAGAAAAATAATATCCCATCCTAATTTACGAGCATGTAG
oligo160	GTATGGGATTTTGCTAAACAACTTTCAACAGTTTCAGCGGAGTGAG
oligo161	ACTAAAGGAATTGCGAATAATAATTTTTTCACGTTGAAAATCTCCA
oligo162	CTTAGCCGGAACGAGGCGCAGACGGTCAATCATAAGGGAACCGAAC
oligo163	ACGGAGATTTGTATCATCGCCTGATAAATTGTGTCGAAATCCGCGA
oligo165	GAACGAGTAGATTTAGTTTGACCATTAGATACATTTCGCAAATGGT
oligo166	GCTATATTTTCATTTGGGGCGCGAGCTGAAAAGGTGGCATCAATTC
25b_BioAdapt_PB	/5BiotinTEG/TTT TGCCCAGACACGTTAACCAGAG
25b_ND_OvH1_PB	ACGGAGGGTAGAAGCCCTTTTTAAGAAAAGTAA TT CTCTGGTTAACGTGTCTGGGCA
25b_ND_OvH2_PB	GAACCGCGCCATCTTTTCATAATCAAAATCACC TT CTCTGGTTAACGTGTCTGGGCA
25b_ND_OvH3_PB	GGAGGTCAGTTGGCTTTTGATGATACAGGAGTGTAC TT CTCTGGTTAACGTGTCTGGGCA
25b_ND_OvH4_PB	TAAATATGTAAAATGTTTAGACTGGATAGCGTC TT CTCTGGTTAACGTGTCTGGGCA
ND_Mag_Twzr_Tether1_BP	GCGCCATTGCAACAGGAAAAACTTTTCGTGCGCACCCAGGTAGACAAGGACAACT
Constrained Helix 0	GAT TTA GAA GTA TTA GAC TTT ACA AAC AAT TCG ACA ACT CGT ATT GGT TAT CTA AAA TAT CTT TAG GAG CAC TAA CAA CTA ATA GAT TAG

Constrained Helix 3	TCC GGC TTA GGT TGG GTT ATA TAA CTA TAT GTA AAT GCT GAT GCA GAT TAA GAC GCT GAG AAG AGT CAA TAG TGA ATT TAT CAA AAT CAT
Constrained Helix 9	GGA ATT GCG AAT AAT AAT TTT TTC ACG TTG AAA ATC TCC AAA AAA TTT CTG TAT GGG ATT TTG CTA AAC AAC TTT CAA CAG TTT CAG CGG
Constrained Helix 12	CGG AAC GAG GCG CAG ACG GTC AAT CAT AAG GGA ACC GAA CTG ACC GTA CAA CGG AGA TTT GTA TCA TCG CCT GAT AAA TTG TGT CGA AAT
Constrained Helix 6	GTC TTT CCT TAT CAT TCC AAG AAC GGG TAT TAA ACC AAG TAC CGC TAA GTC CTG AAC AAG AAA AAT AAT ATC CCA TCC TAA TTT ACG AGC
Constrained Helix 15	TTT TCA TTT GGG GCG CGA GCT GAA AAG GTG GCA TCA ATT CTA CTA TCT GCG AAC GAG TAG ATT TAG TTT GAC CAT TAG ATA CAT TTC GCA

caDNAno Helix # = Loop #	Scaffold sequence of loops (5’-3’)
**Helix 0 = loop 1**	AA TAC GAG TTG TCG AAT TGT TTG TAA AGT CTA ATA CTT CTA AAT CCT CAA ATG TAT TA TCT ATT GAC GGC TCT AAT CTA TTA GTT GTT AGT GCT CCT AAA GAT ATT TTA GAT AAC C
**Helix 3 = loop 2**	TG CAT CAG CAT TTA CAT ATA GTT ATA TAA CCC AAC CTA AGC CGG AGG TTA AAA AGG TA GTC TCT CAG ACC TAT GAT TTT GAT AAA TTC ACT ATT GAC TCT TCT CAG CGT CTT AAT C
**Helix 6 = loop 3**	GC GGT ACT TGG TTT AAT ACC CGT TCT TGG AAT GAT AAG GAA AGA CAG CCG ATT ATT GA TTG GTT TCT ACA TGC TCG TAA ATT AGG ATG GGA TAT TAT TTT TCT TGT TCA GGA CTT A
**Helix 9 = loop 4**	TT TTT TGG AGA TTT TCA ACG TGA AAA AAT TAT TAT TCG CAA TTC CTT TAG TTG TTC CT TTC TAT TCT CAC TCC GCT GAA ACT GTT GAA AGT TGT TTA GCA AAA TCC CAT ACA GAA A
**Helix 12 = loop 5**	GG TCA GTT CGG TTC CCT TAT GAT TGA CCG TCT GCG CCT CGT TCC GGC TAA GTA ACA TG GAG CAG GTC GCG GAT TTC GAC ACA ATT TAT CAG GCG ATG ATA CAA ATC TCC GTT GTA C
**Helix 15 = loop 6**	TA GTA GAA TTG ATG CCA CCT TTT CAG CTC GCG CCC CAA ATG AAA ATA TAG CTA AAC AG GTT ATT GAC CAT TTG CGA AAT GTA TCT AAT GGT CAA ACT AAA TCT ACT CGT TCG CAG A

Supplementary Table S6. Table of the 6 loop sequences from which the force-sensing strands are designed.

Starting Temperature (°C)	Ending Temperature (°C)	Time spent (min) at each interval of 1°C
65	65	30
64	62	60
61	59	120
58	46	180
45	40	60
39	24	30
24	4	1

Supplementary Table S7. 2.5 day thermal annealing ramp for the base ND structure. ND samples are subjected to the following incubation cycles in order to initially disrupt any non-specific base-paring interactions and them slowly cool over ~2.5 days allowing the ND to reach its lowest energy configuration.

Supplementary Methods.
For the creation of our model, we split the design into two main components (see Supplementary Fig. S7). The first is the dsDNA tether, which we model as a worm like chain of contour length L_0=*1055.4nm* with persistence length P=*50nm*. Its force-extension behavior is thus given by

$$F = \frac{k_B T}{P}\left(\frac{1}{4}\left(1 - \frac{z_{WLC}}{L}\right)^{-2} - \frac{1}{4} + \frac{z_{WLC}}{L}\right) \quad (1)$$

where k_B is the Boltzmann constant, and T is the absolute temperature.

The other component is the NanoDyn on the magnetic bead. We assume that the magnetic bead is held fixed by the magnetic field and model the NanoDyn as a stiff rod of length l. We do not know on which point of the bead the attachment between the bead and the NanoDyn is located and denote the unknown angle between the horizontal and the line from the center of the bead to the attachment point of the NanoDyn as Θ_0. We then assume that the NanoDyn is oriented radially away from the bead in the absence of a force but that the attachment between the NanoDyn and magnetic bead has some ability to bend. We thus model the connection between the NanoDyn and the bead as a torsional spring with spring constant K_θ while we denote the actual angle between the horizontal and the axis of the NanoDyn as Θ. In order to avoid numerical instabilities stemming from the fact that a fixed NanoDyn length l results in a maximum extension of the entire system reached for $\Theta = 90°$, we artificially allow the NanoDyn length l to vary by treating the NanoDyn extension as a very stiff spring with rest length l_0=*100*nm and a very stiff spring constant K_l=*10*^{4} pN/nm. This in principle allows arbitrary extensions while still keeping the actual length l very close to its equilibrium value l_0 for any reasonable force. With all these parameters, the extension of the NanoDyn in the vertical direction is

$$z_{tor} = l\sin(\theta) - l_0\sin(\theta_0) \quad (2)$$

and the potential energy of this system is given by

$$V(l,\theta) = \frac{K_l}{2}(l - l_0)^2 + \frac{K_\theta}{2}(\theta - \theta_0)^2 - F \cdot z_{tor}.$$

In equilibrium, this potential energy will be minimized with respect to l and φ and we can thus take the derivative with respect to these two dynamic degrees of freedom separately to yield

$$F = \frac{K_l(l - l_0)}{\sin(\theta)} \quad (3)$$

$$F = \frac{K_\theta(\theta - \theta_0)}{l\cos(\theta)} \quad (4).$$

We can lastly represent the full length of the system by the sum of its components:

$$z = z_{tor} + z_{WLC} + z_0, \quad (5)$$

where z_0 is an unknown offset required since the instrument can only measure relative distances. This gives us five equations Eqs. (1-5) for the five unknowns z_{tor}, z_{WLC}, l, F,and Θ. We can simplify this system of equations somewhat by eliminating the force between equations (1), (3), and (4) to leave us with four equations and unknowns. This set of four equations allows us to numerically solve for the four unknowns using the python function optimize.root for any experimentally measured z and any given set of the parameters z_0, Θ_0, and K_θ, where we restrict the possible angles Θ for the torsional spring to the interval between -π/2 and π/2. Once the four unknowns are determined, we calculate the force F based on Eq. (3) or (4) (choosing Eq. (4) for values of Θ far away from zero and Eq. (5) for values of Θ close to zero to maintain numerical stability). This numerically yields a force-extension curve *F(z)* for a given set of parameters z_0, Θ_0, and K_θ. Finally, we use least squares fitting (python function curve_fit) to find the parameters z_0, Θ_0, and K_θ that minimize the difference between the experimental data and the numerically calculated force extension curve.

To then fit this model to force extension curves featuring a rupture event, additional steps are needed. We first need to find the size of the rupture and the rupture force. We calculate the rupture force as described above. We then take the differences between each consecutive extensions z and use our previously found rupture force to find the region just as the rupture begins (the first data point where the force exceeds the rupture force) and

record the points corresponding to a set of at least 20 consecutive differences larger than 20nm and iterating on this until the following difference goes below the 1nm threshold. We can then use this set of large differences and subtract its sum (the total difference in extension across the entire rupture region) from the high force regime data. For all data points in the rupture region itself the extension is set to the extension at the beginning of the rupture region. Then, a force extension curve is fitted as described above to the data, from which the rupture has been removed. In the absence of a detailed understanding of the mechanics of the flexible part of the open NanoDyn, we finally reintroduce the rupture by extending the tether for extensions beyond the rupture point. As we know that only a fraction of the total length of the tether is contributing to the extension of the system at a given finite force, we must again numerically solve the force-extension equation of the worm like chain

$$F_{rup} = \frac{k_b T}{P}\left(\frac{1}{4}\left(1-\frac{\Delta z}{\Delta L}\right)^{-2} - \frac{1}{4} + \frac{\Delta z}{\Delta L}\right)$$

to obtain the extension change ΔL of the worm like chain required to result in a Δz jump of the measured extension at the rupture force F_{rup}.

The values of the fit parameters Θ_0 and K_Θ are shown in Figure S7b. Θ_0 varies between 0 and -π/2 with a preponderance of values closer to 0 as appropriate for random attachments anywhere on the spherical surface of the magnetic beads. The fit values of K_Θ fall around $5 \cdot 10^4$ pN nm/rad, which at a diameter of the cylindrical cross-sections of the device translates into a linear spring constant on the order of 10^2 pN/nm of the dsDNA attachments. This being two orders of magnitude softer than the eV/Å^2=10^4 pN/nm spring constant of a typical covalent bond seems plausible given that the mechanical stability of the dsDNA attachments is due to hydrogen bonding of the base pairs.

To fit a model to an entire force-extension curve without removing the rupture event, we identify the data points in the rupture event as described above but rather than setting them to the extension at the beginning of the rupture region, remove them altogether and split the remaining data into a pre-rupture and a post-rupture part. We then simultaneously fit the expression above to the pre-rupture part and a modified expression to the post-rupture part. As the first modification for the post-rupture part, we leave the rest length of the (remaining) NanoDyn l_0 as an additional fit parameter (it is fixed at $l_0=100nm$ for the pre-rupture part). As the second modification, we change the length of the worm like chain modeling the tether from $L=L_0=1055.4nm$ to $L=L_0+\Delta L$, where ΔL is yet another fit parameter meant to capture the mechanical behavior of the opened connectors. This fit is performed for all data sets that contain a rupture event (see Figure S19).
